# Correction: Inflammatory serum factors from aortic valve stenosis patients modulate sex differences in valvular myofibroblast activation and osteoblast-like differentiation

**DOI:** 10.1039/d2bm90082c

**Published:** 2022-10-25

**Authors:** Brandon J. Vogt, Kristi S. Anseth, Brian A. Aguado

**Affiliations:** Department of Bioengineering, University of California San Diego La Jolla CA 92093 USA baguado@eng.ucsd.edu; Sanford Consortium for Regenerative Medicine La Jolla CA 92037 USA; Department of Chemical and Biological Engineering, University of Colorado Boulder CO 80303 USA; BioFrontiers Institute, University of Colorado Boulder CO 80309 USA

## Abstract

Correction for ‘Inflammatory serum factors from aortic valve stenosis patients modulate sex differences in valvular myofibroblast activation and osteoblast-like differentiation’ by Brandon J. Vogt *et al.*, *Biomater. Sci.*, 2022, https://doi.org/10.1039/d2bm00844k.

The authors regret the incorrect inclusion of an author in the original manuscript. The corrected authorship list is as shown above.

The Royal Society of Chemistry apologises for these errors and any consequent inconvenience to authors and readers.

## Supplementary Material

